# CTRP-3 Regulates NOD1-mediated Inflammation and NOD1 Expression in Adipocytes and Adipose Tissue

**DOI:** 10.1007/s10753-021-01497-w

**Published:** 2021-06-24

**Authors:** Andreas Schmid, Andreas Schäffler, Thomas Karrasch

**Affiliations:** Department of Internal Medicine III, University Hospital of Giessen, Giessen, Germany

**Keywords:** C1q/TNF-related protein-3, adipocyte, macrophage, adipose tissue, NOD1, peptidoglycan

## Abstract

The anti-inflammatory adipokine CTRP-3 might affect innate immune reactions such as NOD1. The impact of CTRP-3 on NOD1-mediated inflammation in adipocytes and monocytic cells as well as on NOD1 expression was investigated. Murine 3T3-L1 pre-adipocytes and adipocytes as well as human THP-1 monocyte-like cells were co-stimulated with the synthetic NOD1 agonist Tri-DAP and recombinant CTRP-3. Gonadal adipose tissue and primary adipocytes were obtained from a murine model carrying a knockout (KO) of CTRP-3 in adipocytes but not in stroma-vascular cells. Wildtype mice with lipopolysaccharide (LPS)-induced elevated NOD1 expression were treated with CTRP-3. Secreted inflammatory cytokines in cell supernatants were measured by ELISA and mRNA levels were quantified by RT-PCR. Pro-inflammatory chemokine and cytokine secretion (MCP-1, RANTES, TNFα) was induced by NOD1 activation in adipocytes and monocyte-like cells, and MCP-1 and RANTES release was effectively inhibited by pre-incubation of cells with CTRP-3. CTRP-3 also antagonized LPS-triggered induction of NOD1 gene expression in murine adipose tissue, whereas adipocyte CTRP-3 deficiency upregulated NOD1 expression in adipose tissue. CTRP-3 is an effective antagonist of peptidoglycan-induced, NOD1-mediated inflammation and of LPS-induced NOD1 expression. Since basal NOD1 expression is increased by adipocyte CTRP-3 deficiency, there have to be also inflammation-independent mechanisms of NOD1 expression regulation by CTRP-3.

## INTRODUCTION

C1q/TNF-related protein (CTRP)-3 is a family member of adiponectin paralogs (CTRP1-15) [1] that is mainly expressed in adipose and gonadal tissues and in kidney [2]. It represents an adipokine with pleiotropic functions, including regulation of cell proliferation in different cell types [3, 4] and predominantly beneficial metabolic and immunomodulatory effects [5–7]. Of note, previous studies have revealed the role of CTRP-3 as an endogenous LPS antagonist in adipose tissue and inhibitor of toll-like receptor (TLR)4-mediated inflammation in adipocytes and monocytes *in vitro* [6, 8]. Furthermore, application of exogenous, recombinant CTRP-3 attenuated both local (adipose tissue) and systemic inflammation in a murine model of LPS-induced systemic inflammatory response syndrome (SIRS) [9]. Due to its inhibitory impact on pro-inflammatory TLR4 signaling, CTRP-3 has to be regarded as an adipokine linking metabolism, inflammation, and immunity [10], especially in the context of “metaflammation” [11].

In innate immunity, mechanisms of host defense against tissue damage and exogenous pathogens are largely based on various classes of pattern recognition receptors (PRRs) [12]. These receptors recognize microbial and pathogen-associated molecular patterns (PAMPs) as well as endogenous signals released by damaged cells, the so-called damage-associated molecular patterns (DAMPs) [13, 14]. PRRs include TLRs, C-type lectin receptors (CLRs), retinoid acid-inducible gene-I-like receptors (RLRs), and NOD-like receptors (NLRs). While TLRs and CLRs represent transmembrane receptors with predominant plasma membrane and endosomal localization, RLRs and NLRs are mainly localized in cytoplasm [14]. Upon recognition of PAMPs or DAMPs, PRRs in immune cells induce a predominantly pro-inflammatory gene expression profile, including cytokines, chemokines, interferons, and anti-microbial peptides.

Most of the TLRs are expressed in adipocytes, and recent research has also provided evidence of functional TLR signaling with impact on adipocyte expression of immunomodulatory factors [15–17]. Besides TLRs, the role of the NLR nucleotide binding oligomerization domain containing 1 (NOD1) in fat cell biology and adipose tissue physiology has recently gained increasing attention. NOD1 recognizes fragments of bacterial peptidoglycans [18] and upon stimulation it mediates chemokine expression with subsequent immune cell recruitment [19]. Previous studies have shown promotion of adipose tissue inflammation, cellular insulin resistance [20], and impaired adipocyte differentiation [21] upon NOD1 activation. NOD1 stimulation by peptidoglycans has also been reported to induce adipocyte lipolysis [22], subsequently promoting inflammation by diacylglycerol accumulation [23]. Furthermore, Zhou et al. described increased NOD1 activity to be associated with the metabolic syndrome, thus representing a potential machinery of metabolic inflammation and development of insulin resistance [24].

Data on potential regulatory interrelations between immunomodulatory adipokines and NOD1 signaling are scarce. The hypothesis of the present study is based on the question whether CTRP-3 regulates adipocyte NOD1 expression and NOD1-mediated inflammatory gene expression. Therefore, it was the aim of the present study:

- to investigate the effects of NOD1 activation on MCP-1/RANTES/TNFα expression in murine pre-adipocytes and mature adipocytes as well as in human monocyte-like THP-1 cells,
- to study the effect of recombinant CTRP-3 on LPS-induced NOD1 expression *in vivo*,
- to elaborate whether deficiency of CTRP-3 in adipocytes affects basal NOD1 expression in total adipose tissue and in primary adipocytes.

## MATERIALS AND METHODS

### Cell Culture Experiments

#### Murine 3T3-L1 Pre-adipocytes and Mature Adipocytes

3T3-L1 pre-adipocytes [25] were cultured and differentiated into mature adipocytes as described recently [26]. Briefly, cells were cultured at 37 °C and 5 % CO_2_ in Dulbecco’s Modified Eagle Medium (Biochrom AG, Berlin, Germany) supplemented with 10 % newborn calf serum (Sigma-Aldrich, Deisenhofen, Germany) and 1 % penicillin/streptomycin (Aidenbach, Germany) and were differentiated into adipocytes in DMEM/F12/glutamate medium (Lonza, Basel, Switzerland) supplemented with 20 μM 3-isobutyl-methyl-xanthine (Serva, Heidelberg, Germany), 1 μM corticosterone, 100 nM insulin, 200 μM ascorbate, 2 μg/ml transferrin, 5 % fetal calf serum (FCS, Sigma-Aldrich, Deisenhofen, Germany), 1 μM biotin, 17 μM pantothenate, 1 % penicillin/streptomycin (all from Sigma Aldrich, Deisenhofen Germany), and 300 μg/ml Pedersen fetuin (MP Biomedicals, Illkirch, France) [27, 28]. A differentiation protocol reported in the literature [25, 29–32] was used with slight modifications, with light microscopy control of adipocyte phenotype. Mature adipocytes were incubated under serum-free conditions prior to stimulation experiments. The synthetic NOD1 agonist L-Ala-γ-D-Glu-mDAP (Tri-DAP) [20] (10 ng/ml; Invivogen, San Diego, CA, USA) and recombinant CTRP-3 (expressed in insect cells, see below) were used for co-stimulation experiments (incubation time 18 h). Cells were pre-incubated with CTRP-3 for 30 min prior to Tri-DAP treatment. The applied doses of Tri-DAP and CTRP-3 did not impair cell viability as had been tested prior to stimulation experiments. In order to exclude unexpected effects on cell viability, LDH (lactate dehydrogenase) activity was measured in cell supernatants (Cytotoxicity Detection Kit, Roche, Mannheim, Germany).

#### THP-1 Monocyte-like Cells

THP-1 cells (from American Type Culture Collection; Rockville, MD, USA) were cultured at 37 °C and 5 % CO_2_ in RPMI 1640 medium (Sigma-Aldrich, Deisenhofen, Germany) supplemented with 10 % fetal calf serum (FCS; Biochrom AG, Berlin, Germany) and 1 % penicillin/streptomycin (PAN, Aidenbach, Germany). To induce monocyte to macrophage differentiation, THP-1 cells were cultured in the presence of 160 nM phorbol 12-myristate 13-acetate (PMA; Sigma-Aldrich, Deisenhofen, Germany). Macrophages were incubated with serum-free RPMI 1640 medium for stimulation experiments. The synthetic NOD1 ligand Tri-DAP (10 ng/ml) and recombinant CTRP-3 were used for co-stimulation experiments (incubation time 18 h). Macrophages were pre-incubated with CTRP-3 for 30 min prior to Tri-DAP treatment. The applied doses of Tri-DAP and CTRP-3 did not impair cell viability as had been tested prior to stimulation experiments. LDH activity was measured in cell supernatants.

#### Quantification of Secretory Protein Concentrations in Cell Culture Supernatants

**Fig. 1:**
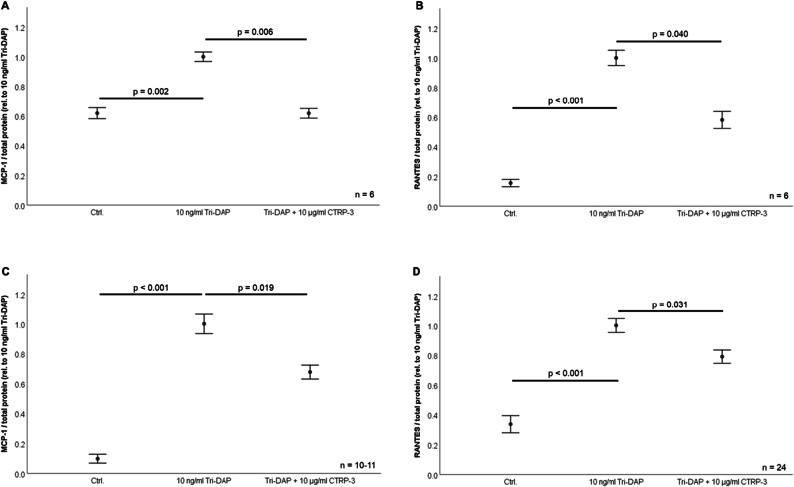
Impact of CTRP-3 on NOD1 mediated pro-inflammatory cytokine release from pre-adipocytes and mature adipocytes. 3T3-L1 fibroblasts/pre-adipocytes as well as mature adipocytes were pre-incubated with 10 μg/ml recombinant CTRP-3 (30 min) prior to overnight (18 h) treatment with 10 ng/ml Tri-DAP. MCP-1 and RANTES protein concentrations in cell supernatants were quantified by ELISA. CTRP-3, C1q/TNF-related protein-3; TD, Tri-DAP. **A** CTRP-3 inhibits Tri-DAP-induced MCP-1 release from 3T3-L1 fibroblasts/pre-adipocytes (n = 6). **B** CTRP-3 inhibits Tri-DAP-induced RANTES release from 3T3-L1 fibroblasts/pre-adipocytes (n = 6). **C** CTRP-3 inhibits Tri-DAP-induced MCP-1 release from mature 3T3-L1 adipocytes (n = 10–11). **D** CTRP-3 inhibits Tri-DAP-induced RANTES release from mature 3T3-L1 adipocytes (n = 24).

**Fig. 2:**
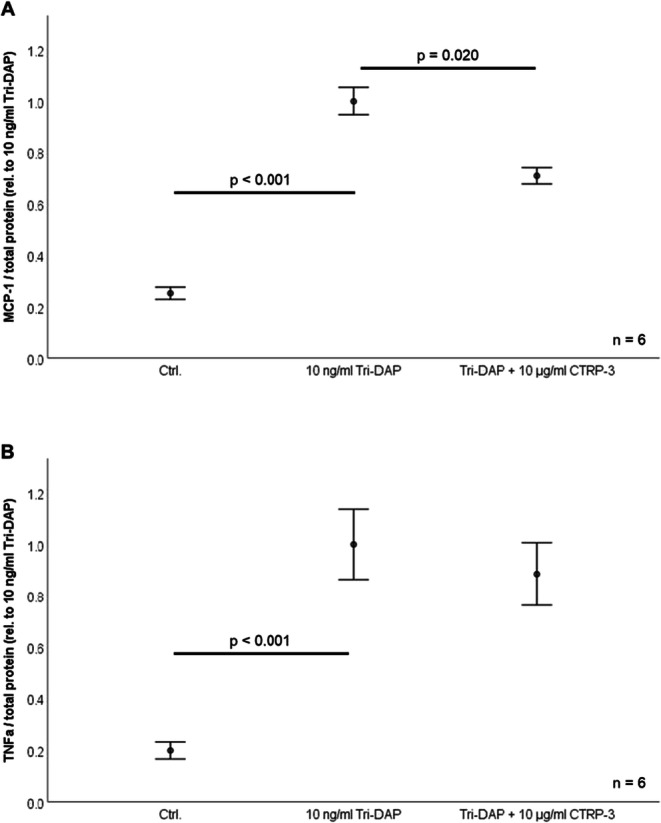
Inhibition of NOD1 mediated pro-inflammatory cytokine release from THP-1 cells. Monocyte-like THP-1 cells were pre-incubated with 10 μg/ml recombinant CTRP-3 (30 min) prior to overnight (18 h) treatment with 10 ng/ml Tri-DAP. MCP-1 and RANTES protein concentrations in cell supernatants were quantified via ELISA. CTRP-3, C1q/TNF-related protein-3; TNFα, tumor necrosis factor α; TD, Tri-DAP. **A** CTRP-3 inhibits Tri-DAP-induced MCP-1 release from THP-1 macrophages (n = 6). **B** Tri-DAP induced TNFα release from THP-1 monocyte-like cells pre-incubated with CTRP-3 (n = 6).

Concentrations of cytokines in cell supernatants were measured in duplicates by ELISA (murine MCP-1: Biolegend; San Diego, USA; murine RANTES, human MCP-1, human TNFα: DuoSet ELISA development systems, R&D Systems, Wiesbaden, Germany). In Figs. 1 and 2, chemokine/cytokine concentrations in cell culture supernatants are expressed as relative values, and mean values ± standard error of the mean (SEM) are given. Measurement was generally repeated for exceptional samples exceeding a duplicate variation of 20%. The lower detection limits were 15.6 pg/ml for human MCP-1 and TNFα, 31.2 pg/ml for murine RANTES, and 62.5 pg/ml for murine MCP-1.

#### Adipose Tissue and Adipocyte mRNA Extraction

Total RNA was isolated from adipose tissue and cells as reported recently [26]. Briefly, cells and tissue were homogenized in TRIzol® Reagent (Life Technologies GmbH, Darmstadt, Germany) in combination with gentleMACS dissociator and M-tubes (Miltenyi Biotec GmbH, Bergisch Gladbach, Germany) for dissociation and RNA was isolated applying RNeasy® Mini Kit (Qiagen, Hilden, Germany) including DNase digestion (RNase-Free DNase Set, Qiagen, Hilden, Germany).

#### Quantitative Real-time PCR Analysis of NOD1 mRNA Expression

For gene expression analysis, reverse transcription of RNA (QuantiTect Reverse Transcription Kit from Qiagen, Hilden, Germany) was performed to generate corresponding cDNA for real-time PCR (RT-PCR) (iTaq Universal SYBR Green Supermix, CFX Connect RT-PCR system; Bio-Rad, Munich, Germany). NOD1 mRNA levels in murine adipocytes and adipose tissue were quantified using the following primer sequences:

- Murine NOD1: 5′-CGCTACGGCATTTATGGCTG-3′/5′-CGTCCGTCAGAAGCAGATGA-3′
- Murine GAPDH: 5′-TGTCCGTCGTGGATCTGAC-3′/5′-AGGGAGATGCTCAGTGTTGG-3′

Expression levels of NOD1 were normalized to gene expression of murine GAPDH. All oligonucleotides used were purchased from Metabion, Martinsried, Germany.

#### Recombinant Expression of CTRP-3

Recombinant CTRP-3 protein expression was performed in H5 insect cells (Invitrogen, Karlsruhe, Germany) using the BacPak Baculovirus Expression System (BD Biosciences, Palo Alto, CA, USA) as published earlier [33]. Unlike prokaryotic expression systems, recombinant expression in insect cells usually maintains glycosylation and phosphorylation. Our expression system was proven to generate trimeric CTRP-3 [33]. High purity of recombinant CTRP-3 protein in the preparation was verified by sodium dodecyl sulfate polyacrylamide gel electrophoresis.

### Animals

#### LPS-induced Systemic Inflammatory Response Syndrome (SIRS) Model

In male C57BL/6 wildtype mice (age 8–12 weeks; from Charles River, Sulzfeld, Germany), moderate inflammation was induced by intraperitoneal (i.p.) injection of lipopolysaccharide (LPS; 1 μg per animal) as described recently [26]. The animals were euthanized 2 h after LPS injection and intra-abdominal adipose tissue specimens were resected for gene expression analysis. This animal study was performed at the University of Regensburg, Germany, and was approved by the local government agency (*Regierungspraesidium Oberpfalz*, No. 54-2532.1-14/10).

#### Tissues from CTRP-3 Knockout Mice

Transgenic mice with an adipocyte-specific CTRP-3 knockout (full nomenclature: B6NTac.Cg-*C1qtnf3*^*tm3113Arte*^Tg(*Fabp4-cre*)1Rev; abbreviation: CTRP-3 KO) together with littermate control mice (B6NTac.Cg-*C1qtnf3*^*tm3113Arte*^) were bred under standard conditions and were euthanized for organ and tissue resection. In this novel mouse model, CTRP-3 is completely lacking in mature adipocytes but not in the stroma-vascular cell fraction of adipose tissue. Intra-abdominal adipose tissue specimens were resected and were either used for primary cell isolation or otherwise shock-frosted in liquid nitrogen. Deletion of exon 4 and subsequent frame-shift mutations within the *C1qtnf3* gene were introduced applying the *Cre/loxP* system, resulting in a dysfunctional gene product. Cell-type specificity of the knockout was achieved by transcriptional control of the *Cre* recombinase encoding transgene by the *aP2*-promotor (from *Fabp4* gene). B6NTac.Cg-*C1qtnf3*^*tm3113Arte*^ mice carrying *C1qtnf3* gene alleles with *loxP*-flanked exon 4 were created in collaboration with Taconic Artemis (Cologne, Germany). B6.Cg-Tg(*Fabp4-cre*)1Rev/J mice (purchased from Jackson Laboratories) were back-crossed to C57BL/6NTac genetic background applying the Speed Congenics method for at least 6 generations, resulting in the strain B6.Cg-Tg(*Fabp4-cre*)1Rev/N. This offspring was crossed with C57BL/6NTac-C1qtnf3tm3113Arte mice to generate B6NTac.Cg-*C1qtnf*^*3tm3113Arte*^Tg(*Fabp4-cre*)1Rev mice with adipocyte CTRP-3 KO.

#### Isolation and Cell Culture of Primary Murine Adipocytes

For primary cell culture, fresh intra-abdominal adipose tissue obtained from CTRP-3 KO mice was cut into small pieces and treated with 0.225 U/ml collagenase NB6 (Serva) at 37 °C for a maximum of 60 min. Digestion process was stopped by adding twice the amount of buffer (PBS containing 0.5% BSA and 2 mM EDTA). Cell suspension was filtered by 120-μm nylon mesh to eliminate undissolved tissue. Pre-adipocytes were separated from adipocytes by 10 min centrifugation at 300 *g* and 4 °C. Magnetic labeling plus depletion of non-adipocyte progenitor cells was done according to the manufacturer’s instructions (Adipose Tissue Progenitor Isolation Kit mouse, MACS Miltenyi Biotec, Bergisch Gladbach, Germany), as well as magnetic labeling and positive selection of adipocyte progenitor cells. Isolated pre-adipocytes were seeded at a density of 2.03 × 10^4^ cells/cm^2^ in DMEM (Dulbecco’s Modified Eagle Medium, Biochrom AG, Berlin, Germany) that was supplemented with 10% newborn calf serum (NCS; from Sigma-Aldrich, Deisenhofen, Germany) and were cultured at 37 °C and 5% CO_2_. Adipocyte differentiation was initiated after cells had reached 85 % confluency. Media for hormonal differentiation were supplemented as described above for 3T3-L1 cell line. Cellular phenotype during adipocyte differentiation was monitored by light microscopy.

### Statistical Analysis

For explorative data analysis, a statistical software package (SPSS 26.0) was used. Non-parametric numerical parameters were analyzed by the Mann-Whitney U-test (for 2 unrelated samples) and the Kruskal-Wallis test (>2 unrelated samples). A p-value below 0.05 (two-tailed) was considered as statistically significant. In the figures, the bars are showing the mean values and the whiskers are giving the standard error of the mean (SEM).

## RESULTS

### CTRP-3 Has Inhibitory Effects on NOD1 Activation in 3T3-L1 Pre-adipocytes and Adipocytes

3T3-L1 fibroblasts/pre-adipocytes were stimulated with Tri-DAP (10 ng/ml) in order to test for a NOD1-mediated inflammatory response. Upon Tri-DAP stimulation, a highly significant increase of MCP-1 (p = 0.002; Fig. 1A) and RANTES (p < 0.001; Fig. 1B) in cell supernatants was observed. Pre-incubation of cells with CTRP-3 (10 μg/ml; 30 min) significantly antagonized the effects of NOD1 activation on MCP-1 (p = 0.006; Fig. 1A) and RANTES (p = 0.040; Fig. 1B) release, whereas CTRP-3 alone had no effect. NOD1 activation by Tri-DAP was also effective in mature and fully differentiated adipocytes regarding MCP-1 and RANTES secretion, respectively (Fig. 1C and D). Tri-DAP induced MCP-1 and RANTES release were abrogated by co-stimulation with 10 μg/ml CTRP-3 (p = 0.019 and p = 0.031, respectively), whereas CTRP-3 alone had no effect.

### CTRP-3 Effects on NOD1 Activation in THP-1 Monocyte-like Cells

THP-1 cells were differentiated to macrophage-like cells by PMA treatment and stimulated with Tri-DAP (10 ng/ml), resulting in increased MCP-1 and TNFα levels in cell supernatants after 18 h (p < 0.001; Fig. 2A and B). The release of MCP-1 was significantly attenuated by pre-incubation with CTRP-3 (10 μg/ml) (p = 0.020; Fig. 2A), whereas TNFα release was only slightly decreased (p = 0.352; Fig. 2B).

### Effects of Intraperitoneal LPS and CTRP-3 Application on NOD1 Gene Expression in Intra-abdominal and Subcutaneous Adipose Tissue in Mice *In Vivo*

**Fig. 3:**
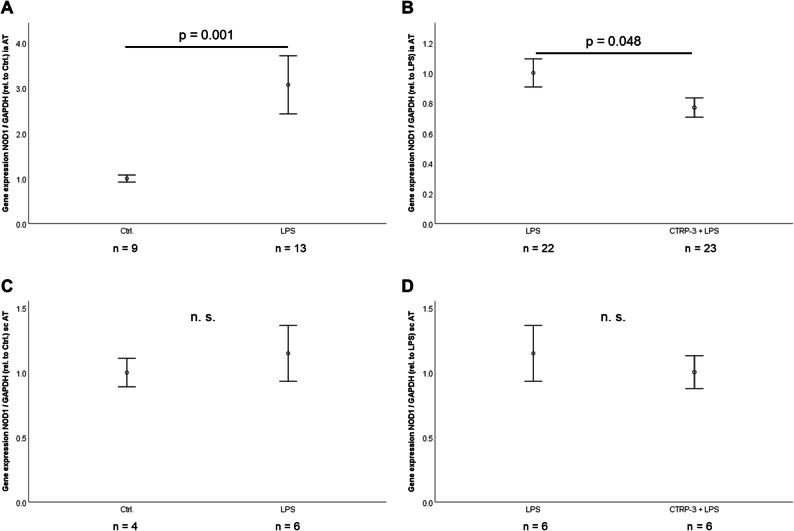
CTRP-3 attenuates LPS-induced NOD1 gene expression *in vivo* in murine adipose tissue. C57BL/6 mice were injected i.p. with recombinant CTRP-3 (10 μg/animal) 30 min prior to i.p. injection of LPS (1 μg/animal). After 2 h, intra-abdominal adipose tissue was obtained post-mortem. NOD1 gene expression levels were quantified by RT-PCR and normalized to GAPDH gene expression. AT, adipose tissue; CTRP-3, C1q/TNF-related protein-3; i.p., intraperitoneal; ia, intra-abdominal; LPS, lipopolysaccharide; sc, subcutaneous; TD, Tri-DAP. **A** LPS induces NOD1 gene expression in intra-abdominal adipose tissue *in vivo* in mice. **B** CTRP-3 attenuates LPS-induced NOD1 gene expression in intra-abdominal adipose tissue in mice. **C** and **D** LPS and CTRP-3 do not affect NOD1 gene expression in subcutaneous adipose tissue in mice.

In C57BL/6 wildtype mice, intraperitoneal (i.p.) LPS injection (1 μg/animal) caused a significant increase of gonadal adipose tissue NOD1 gene expression (p = 0.001; Fig. 3A). This effect was significantly attenuated by pre-treatment of mice with recombinant CTRP-3 (10 μg/animal, i.p. injection) 30 min prior to LPS application (p = 0.048; Fig. 3B). In contrast to these findings, neither LPS alone (Fig. 3C) nor co-treatment with LPS and CTRP-3 (Fig. 3D) had a significant impact on NOD1 expression in subcutaneous adipose tissue.

### Elevated NOD1 Gene Expression in Adipose Tissue and Primary Adipocytes from Mice with Adipocyte-specific CTRP-3 Deficiency

**Fig. 4:**
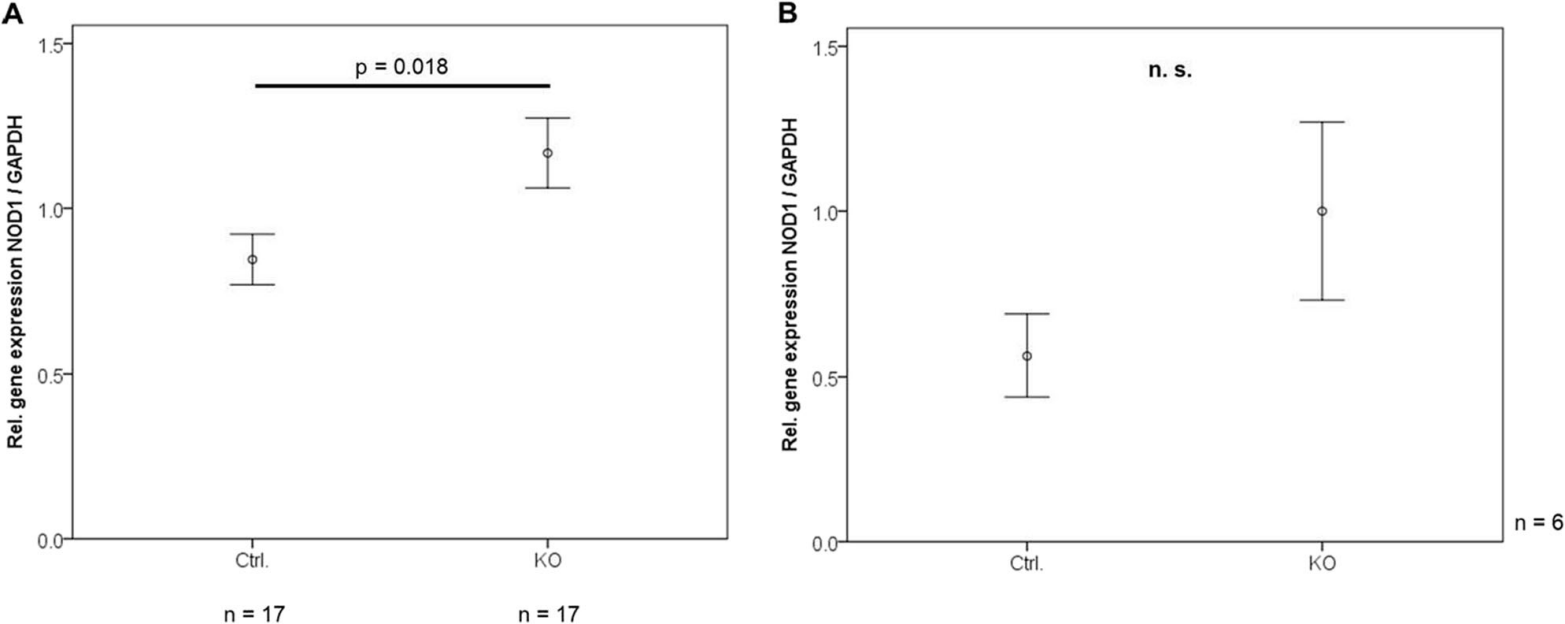
Impact of adipocyte CTRP-3 deficiency on NOD1 gene expression in adipose tissue and primary adipocytes. Intra-abdominal adipose tissue was obtained from control mice and mice with an adipocyte-specific CTRP-3 knockout. Primary pre-adipocytes were derived from intra-abdominal adipose tissue and were differentiated to mature adipocytes *ex vivo*. NOD1 gene expression levels were quantified via RT-PCR and normalized to GAPDH gene expression. Ctrl., control mice; CTRP-3, C1q/TNF-related protein-3; KO, knockout mice; TD, Tri-DAP. **A** NOD1 gene expression levels are elevated in intra-abdominal adipose tissue from female CTRP-3 KO mice (n = 17) when compared to littermate controls. **B** NOD1 gene expression levels are not significantly altered in primary intra-abdominal adipocytes from female CTRP-3 KO mice (n = 6).

Gene expression analysis in intra-abdominal adipose tissue of CTRP-3 KO mice and CTRP-3 positive control animals (littermates) revealed higher NOD1 mRNA levels in KO animals (p = 0.018; Fig. 4A). The promotion of NOD1 transcription in total adipose tissue by adipocyte CTRP-3 deficiency might indicate inhibitory effects of CTRP-3 on basal NOD1 gene expression.

Primary pre-adipocytes were isolated from intra-abdominal adipose tissue of CTRP-3 KO and control mice and were differentiated into fat cells *in vitro*. After 9 days, when cells exhibited a mature adipocyte phenotype, mRNA was isolated and NOD1 expression was quantified. In adipocytes derived from CTRP-3 deficient cells, a considerable, yet non-significant trend of elevated NOD1 mRNA levels was observed (p = 0.065; Fig. 4B).

## DISCUSSION

The role of adipocytes and their secretory proteins in various processes of innate immunity such as TLRs and PRRs is a novel and complex aspect of adipose inflammation or even metaflammation. Regarding the immunomodulatory functions of the adipokine CTRP-3, its role as endogenous LPS antagonist and anti-inflammatory adipokine—at least in adipose tissue—has been described [6, 10]. Outside adipose tissue physiology, general and supra-physiological overexpression of CTRP-3 failed—in contrast—to demonstrate an anti-inflammatory capacity of CTRP-3 [34]. Recent studies have provided evidence of active and functional NOD1 signaling pathways in adipocytes affecting fat cell differentiation, metabolism, and inflammation [20–22], whereas an impact of the closely related NLR NOD2 on adipocyte biology and adipose inflammation seems rather questionable so far [21, 24, 35] and requires further research applying adequate clinical studies and experimental/genetic models in the context of metaflammation. To the best of our knowledge, the present study is the first to investigate CTRP-3 effects on peptidoglycan-triggered and NOD1-mediated inflammation as well as NOD1 gene expression. NOD1 activation by the synthetic diaminopimelic acid derivative Tri-DAP in murine 3T3-L1 fibroblasts/pre-adipocytes as well as in mature adipocytes strongly induced the secretion of the chemokines MCP-1 and RANTES *in vitro*. This pro-inflammatory reaction was significantly attenuated by co-stimulation with exogenous and recombinant CTRP-3. It is reasonable to build the hypothesis that inhibition of NOD1-mediated expression and secretion of chemoattractant proteins by adipocytes might antagonize immune cell recruitment to adipose tissue as well as pro-inflammatory activation of tissue-resident macrophages. In a subsequent experimental approach, human THP-1 cells were stimulated with Tri-DAP, resulting in increased MCP-1 and TNFα release. Similar to the observations in 3T3-L1 adipocytes, the strong induction of MCP-1 secretion was significantly attenuated by co-stimulation of these monocyte-like cells with CTRP-3, whereas TNFα release was not significantly affected. Previous studies have suggested CTRP-3 as an important paracrine, immunomodulatory factor in the context of an inflammatory adipocyte-macrophage cross-talk [10, 36]. The present data suggest that CTRP-3 also affects analogous cellular interactions in peptidoglycan-induced, NOD1-dependent adipose tissue inflammation. Although primarily descriptive, our findings are new and consistent and have been evaluated in human and murine cell lines *in vitro* and *in vivo* by using two sophisticated murine models (LPS-induced SIRS model and a model of CTRP-3 knockout in adipocytes). However, subsequent mechanistic studies are needed to investigate the involved intracellular signal transduction components and pathways of both, NOD1 activation and CTRP-3-induced inhibition.

Besides NOD1 activation, NOD1 expression in adipocytes and adipose tissue has been poorly described so far. During LPS-induced moderate inflammation, mice exhibited strongly increased NOD1 mRNA expression in intra-abdominal adipose tissue. This effect was absent in subcutaneous adipose tissue and therefore appears to be specific for the intra-abdominal fat compartment. In animals that had been treated with recombinant CTRP-3 30 min prior to LPS treatment, elevated intra-abdominal adipose tissue NOD1 expression was significantly attenuated. Vice versa and in accordance with this observation, transgenic mice with adipocyte CTRP-3 knockout exhibited significantly elevated basal NOD1 mRNA levels in intra-abdominal total adipose tissue. Furthermore, there was a considerable, yet non-significant trend of increased NOD1 gene expression in CTRP-3 deficient primary adipocytes. Taken together, CTRP-3 apparently has an inhibitory role in the regulation of basal and stimulated NOD1 gene expression in adipose tissue.

Based on the present data, future experimental approaches will have to gain a more precise insight in mechanisms underlying the antagonizing effects exerted by CTRP-3, including detailed analysis of involved signaling pathway components and genetic models, especially for cell-type-specific NOD1 deficiency. Furthermore, a potential immune-regulatory role and regulatory interaction with CTRP-3 should be investigated for NOD1-related NLRs, such as NOD2, as well as a putative specificity of the observed effects for different adipose tissue compartments.

## CONCLUSIONS

CTRP-3 is an effective antagonist of pro-inflammatory NOD1 activation in adipocytes as well as in monocyte-like cells. Furthermore, it appears to inhibit NOD1 expression in adipocytes and in adipose tissue *ex vivo* and *in vitro*. Future mechanistic studies will have to investigate in detail the pathways of NOD1 activation and CTRP-3 inhibition in adipose tissue in specific disease contexts.

## Data Availability

All data and material included in the present study are available.

## Code Availability

N/A.
